# Remarks upon the Morbid Anatomy of the Brain in Insanity

**Published:** 1851-07-01

**Authors:** Holmes Coote

**Affiliations:** ENGLAND, DEMONSTRATOR OF ANATOMY AT SAINT BARTHOLOMEW'S HOSPITAL


### REMARKS UPON THE MORBID ANATOMY OF THE BRAIN IN INSANITY.

BY HOLMES COOTE, F. R. COLL. SURG. ENGLAND, DEMONSTRATOR OF ANATOMY AT SAINT BARTHOLOMEW'S HOSPITAL.

In the investigation of the pathology of the brain, we must bear in mind that the greater proportion of the morbid appearances found after death, cannot be considered as explaining the phenomena of intellectual disturbance, which characterize the last periods of existence. Any attempt, for example, to connect the different forms of mental alienation with the character, locality, or amount of effusions of fluid within the cranium, would undoubtedly fail, inasmuch as the disturbed state of the sensorial functions depends upon the previous condition of the cerebral circulation, of which such morbid appearances are but the result. The infiltration of fluid into the pia mater, by which it becomes converted into a thick sponge-like texture, which has lost all the delicacy and tenuity proper to that membrane; or, its effusion into the ventricular cavities, is observed as well in those who have died violent and raving, as in the silent, the timid, or the desponding. The rapidity with which the fluid is poured forth, deserves consideration, when the symptoms which occur during life are of a nature to justify our forming an opinion upon the subject; for it is well known that any part of the cerebro-spinal centre will bear with apparent impunity an amount of slowly produced pressure, which, if suddenly exercised, would cause immediate death. It is rare, however, that we have any clue to the time when the amount of secretion in these textures is first morbidly increased: the change from the natural state is so gradual, that the brain bears it without further complications than those already existing. In a patient, examined May, 1851, there was found within the head so great an amount of serous effusion, that it might have been expected some symptoms of compression would have been the result: he had been noisy, talkative, and turbulent, up to the time of his death. In a female, examined April, 1847, there was found a moderate amount of purulent effusion in the sac of the arachnoid, in the pia mater, and in the cavity of the ventricles: she had never been otherwise than silent, morose, and feeble. Speaking of effusions of serum, Dr. Abercrombie remarks, "Whenever this remarkable condition occurs, it naturally becomes the prominent object of attention; and, as it has been by long established usage strongly associated with the idea of pressure upon the brain, the investigation has generally been directed to the discovery of a compressing cause. Effused fluid, having been found, upon examination after death, in a great proportion of the cases referred to, has on this principle been considered as explaining the symptoms, and here probably the investigation closed. This course of inquiry seems to have been the occasion of so much of that obscurity, which so long involved the pathology of affections of the brain."

Although we are unable, with any accuracy, to refer particular symptoms to disease of definite regions of the brain, yet where disorganization or softening of the cerebral substance ensues, it is attended with a marked impairment of the intellectual faculties; and this condition of the mind, in a medico-legal point of view, quite distinct from insanity, is equally deserving of supervision and direction. I cannot believe in any positive destruction or disorganization of the substance of the brain, without some corresponding functional impairment; and I attach but little importance to cases stated to be illustrative of the converse view, when all the proofs of a perfect and vigorous mind consist in the fact, that the patient can answer in a rational manner the commonest questions relative to his own feelings and wants. If the injury to the cerebral substance be limited; if, for example, a small quantity of blood be effused, from the giving way of the coats of some diseased capillary vessels, the reparation may be complete; but the occurrence of the accident is marked by symptoms not to be mistaken, varying from a slight and transient giddiness, to complete paralysis and loss of speech. In course of time the blood becomes disorganized and reduced to a granular mass; the dark spot, where the extravasation occurred, passes through the different shades of brown, yellow, or yellowish white, as the disintegration of the blood-discs is the more complete. A delicate and organised cyst surrounds the torn nerve fibres, which in course of time undergo the same change as the blood-discs, and are removed as a foreign body. In the examination of a brain in which the process of repair may be said to be complete, we find nothing but the thin, transparent and empty cyst, formed of condensed areolar tissue. The patient, though for a long time conscious of a feeling of insecurity in his head, eventually regains his former clearness and vigour. The same, however, cannot be said when the extravasation is considerable, or where small superficial extravasations have recurred in quick succession. The mind, in such cases, becomes permanently weakened, and the patient is, to a considerable extent, at the mercy of those who surround him. The external senses may convey true impressions, and the patient may retain the faculty of reasoning correctly; but he may, at the same time, have lost the vigour of mind requisite to form his own conclusions, and to act independently upon them. Such a person, though clearly not insane, can hardly be said to be competent to undertake the management of his affairs.

Amongst the many causes which disturb the sensorial functions, the most common doubtless is a faulty condition of the circulation. The blood itself, frequently in an abnormal state, is either propelled with too great force, or not in sufficient quantity through the cerebral vessels. In a brain rather below the proper standard of development, these conditions will produce, in a permanent form, symptoms which, in a well-organized brain, would be only transient. In order to express an opinion worthy of attention, as to whether in this class of cases any morbid appearances can be discovered after death, the examination must be continued from the head to the other regions of the body: the state of the lungs and of the heart must be carefully ascertained; that of the liver, alimentary canal, and kidneys, should be duly investigated.

Many who have professed familiarity with these subjects, have asserted that the morbid appearances found in the bodies of the insane were unworthy of record: they should have rather confessed that they were unable to appreciate their value. With the more thorough and complete investigation of these matters, we may hope eventually to arrive at some correct views as to the nature of those laws, the transgression of which leads to sensorial disturbance, but no approach to the truth can be made, except through the portal of morbid anatomy, which has revealed this important fact, that the record of post-mortem examinations, as preserved in an asylum for the insane, differs in most striking and essential particulars from that preserved in a general public hospital.

The cavity of the body which next demands attention is the thorax, containing those important organs, the lungs and the heart.

The frequency of tuberculous deposit in the lungs has been remarked by other writers. There seems to exist, in many families, subject to tuberculosis, an hereditary disposition to insanity. The disease of the brain does not show itself until the occurrence of some of those troubles, through which all have to pass, in greater or in less degree. Disappointment or inability to carry out projected plans excites, in some cases, violent fits of passion, connected with increased activity of the cerebral circulation, terminating occasionally, though rarely, in inflammation; in other cases great melancholy, and depression of spirits, accompanied by general emaciation. The deposit of tubercle appears to have been arrested in many cases. Having formed in the upper part of the lungs, it loses its fluidity and semi-transparency — becomes dull, yellowish, and opaque, and eventually cretaceous, when it is either imbedded in the healthy pulmonary substance, or is coughed up, leaving a dark-coloured, irregular, and puckered cicatrix at the apex of the lung. Such appearances are very common, and they lead to the conclusion that, destructive to life as is the progress of that disease known as consumption, in its acute form, especially when it attacks the young, tubercles are deposited in a very large proportion of persons at some period of life in the structure of the lungs, where, after exciting more or less disturbance, they either undergo the changes above described, or are coughed up in the expectoration, the patient remaining well afterwards, as far as the respiratory organs are concerned.

Miss —— became deranged in consequence of domestic calamities. She died, December, 1847, in an extremely emaciated state. Examination of the body, December 11.

There was considerable opacity and thickening of the arachnoid membrane, with infiltration of fluid into the pia mater. The ventricles contained an increased amount of serous fluid: the cerebral substance presented, upon being cut into, more numerous red points than natural.

The structure of the lungs was generally healthy, but the opposed surfaces of the right pleura were united, to a small extent, by an old adhesion in the upper and posterior part of the thorax. In the adjoining pulmonary substance was a knot of yellowish white concretion, the size of a pea, imbedded in a delicate capsule. The surrounding structures were puckered and drawn in, but otherwise healthy; there was no other trace of tubercle.

The condition of the brain, however, not uncommonly seems, for the time, to render a patient exempt from those sufferings usually attendant upon tuberculous deposit. Disease goes on, without destroying life, to a greater extent than is observed in general practice. I have occasionally examined lungs in which, from the general consolidation by tuberculous deposit, it has been a source of inquiry what parts have been subservient to the purposes of respiration. Both sides of the chest were occupied by heavy, incompressible masses, which, when divided, presented a continuous surface of yellowish white colour, speckled with black, or blackish grey lines and spots. Patients in this state are usually feeble; they remain either in bed, or are confined to a room, where, being always at rest, there is no call for any increased respiratory acts.

An old woman died in Bethlem Hospital, April, 1850. Having been for many years spiteful and vindictive, she suddenly, and without obvious cause, became good-tempered and talkative. Body examined April 30.

She was extremely emaciated: the skull-cap was thin, light, and shallow; the arachnoid membrane was transparent, but separated from the cerebral convolutions, which were much shrunken, by the effusion of a large quantity of clear serous fluid in the layers of the arachnoid. The shrinking and atrophy of the convolutions was such as to leave many spaces, which would readily admit the introduction of the end of the finger. The cerebral substance was firm, but vascular.

Both lungs were universally infiltrated by the deposit of light grey semi-transparent miliary tubercles; there was no softening, nor any trace of a cavity in any part; the whole pulmonary substance seemed occupied by this morbid substance, and sunk when immersed in water, unless the section were taken from the inferior border of the lung, where there were a few dilated air-cells. There were a few old adhesions of the pleura on both sides of the chest; on the right side, the lung was united to the fifth rib by a tough band, which, when divided, was found to form the limit of a small cavity containing a bit of dead bone connected with the rib. Along the course of the ilium were numerous round ulcers with raised margins, which had, doubtless, been the result of the deposit of tubercle.

The mesenteric glands were slightly enlarged.

The uterus was much elongated, and considerably larger than natural in the unimpregnated state. Under its peritoneal covering there was a large collection of yellowish white masses of tubercle, varying in size from a pin's-head to a pea, or even larger; the walls of the uterus in their whole thickness, were occupied by a similar deposit, whilst its cavity was lined by a continuous layer of it, several lines in thickness, and of the consistence of cheese. It closely resembled, in its general characters, as well as in its minute structure, that yellow tubercle found in solid opaque masses in the substance of the testicle, and in the lumbar glands. At the neck of the uterus there was softening and ulceration; the surface, broken, shreddy, and uneven, was covered by a considerable quantity of yellow, purulent matter, mixed with blood. Both ovaries were diseased by the deposit of similar tubercle; the right adhered to the uterus, the left to the sigmoid flexure of the colon.

I believe it to be an almost invariable law, amongst the inhabitants of a country with a climate similar to that of England, that tubercle is deposited first in the lungs. If patients ultimately die from the development of the disease in other situations, still the lungs have first experienced the disease. In the case here stated, it must be confessed that the evidence goes far to prove the uterus to have been the first affected. The amount of tubercle there collected, its infiltration through the walls of the organ, the ulceration going on at its neck, are all strikingly in contrast with the condition of the lungs, where the semi-transparent and semi-fluid morbid product had not yet lost its low vitality, but exhibited under the microscope, a structure consisting of well-marked cells, not very different from those of cancer.

Instances are upon the hospital record, where tubercle has been deposited in the bony parietes of the chest, as well as in the viscera. The cancellous texture of the bone becomes infiltrated by this morbid product, the part swells, ulceration ensues, in a precisely similar manner as when a strumous ulcer forms in the soft parts (*e. g.*, the neck, &c.,) for the purpose of throwing off the infiltrated matter, and a large, irregular ulcerated cavity results, to which the term of carious has been applied. Caries may be defined as tuberculous ulceration of bone. The effects of such a disease upon the thoracic viscera when it occurs in the sternum, may be illustrated by the following case:—

*Examination of the body of ——, Bethlem Hospital.*

Bloodvessels of the brain and membranes rather empty. Cellular membrane of the pia mater covering the cerebral hemispheres greatly infiltrated. Five or six ounces of clear, transparent fluid in the lateral ventricles; much fluid also in the basis of the skull. A few convolutions of the cerebral hemispheres slightly shrunken. Pineal gland converted into a thin cyst, equal in size to a horse-bean, containing a clear, light-yellow fluid.

There was caries of the sternum, with an abscess, and the inflammation had extended to the anterior mediastinum. The pericardium was also inflamed. The right pleura was connected to the sternum, and adhered firmly on the inside to the lung. The right lung was connected to the pleura by extensive old adhesions; tuberculated, and contained a vomica about the size of an ordinary orange. A portion of the right lung red and indurated. There was a pint of fluid in the left pleura; old adhesions of the left lung, and on its surface there was the appearance of an indurated and depressed cicatrix. The substance of the lung was here blackish, hard, and contained small deposits of a greyish cheesy matter. The heart was enlarged much beyond its natural size.

As contrasted with the frequency of tuberculous deposit in the chest in the insane, I may again allude to its rarity in the brain or its membranes. There is no region of the body where it is so seldom seen as the cranial cavity; even in those cases where it has been infiltrated to an extreme extent, both amongst the abdominal and thoracic viscera. Pathological collections confirm this statement; for although specimens are to be seen in which the brain or its membranes are the seat of this disease, yet they bear no proportion, in point of number, to specimens illustrative of the same morbid changes in other situations.

The history of the cases shows that the effects of such deposit, during life, vary according to the region affected; but that there is not manifested necessarily any intellectual disturbance. In what manner, then, are we to regard the frequency of phthisis pulmonalis amongst the insane? Is the cerebral circulation insufficient for the healthy exercise of the sensorial functions, owing to the imperfect arterialisation of the blood? There is, I believe, some truth in this statement, although it will not of itself explain all the phenomena now before us. The chief cause exists in the brain itself, whose development, in these cases, must be incomplete, although hitherto we have failed in detecting the deficiency. Upon such a structure it is easy to imagine the effect of the stimulus of unhealthy blood, and to understand that it would not be exempted from the general want of power which characterized the other component parts of such an organism.

The following cases are good instances of the extensive deposition of tubercle:—

*A criminal lunatic, aged* 48, *died January* 20, 1848.

The membranes of the brain were full of blood: the convolutions were flattened; the lateral ventricles were distended by at least five ounces of clear serous fluid. The septum lucidum was softened, thinned, and shreddy in the longitudinal direction; the foramina of Monro were very large; the corpora striata presented a concave surface towards the ventricular cavities. There was yellow sero-purulent infiltration under the arachnoid membrane at the base of the brain, about the pons varolii and the pituitary body. The third ventricle contained much fluid, and the pressure and distention were greatest in the situation of the commissure of the optic nerves. The patient had been blind for some time before death.

The lungs were everywhere studded with tubercles. Some were distinct, semi-transparent, and grey; others of darker colour, more opaque, and confluent; softening had taken place, so as to form small vomicæ, containing a mixture of pus and tubercle, in many situations, in both lungs. The opposed surfaces of both pleuræ were universally adherent.

The peritoneum was studded throughout with deposits of tubercle, varying in size from a pea, to the last joint of the thumb; large confluent masses were accumulated by the round ligament of the liver and the small omentum. The hepatic vessels and the biliary ducts were completely surrounded, pressed upon, and nearly obliterated. There were numerous old thready adhesions between the opposed peritoneal surfaces. The transverse colon adhered to the front surface of the stomach, and to the under surface of the liver.

The peritoneum was in many situations much thicker than natural, and presented a mottled grey appearance from the copious deposit of pigment. There were masses of tubercle upon the surface and in the interior both of the pancreas and the spleen. The former was firmly attached to the duodenum, the walls of which were thickened by the same morbid deposit. All the other viscera were healthy. There were no ulcers along the course of the intestinal canal.

The pancreas is very rarely changed in structure. This is the only instance in which I have seen it diseased.

*Examination of the body of ——, Criminal Lunatic, Bethlem Hospital, May* 19, 1851.

Skull-cap shallow; dura mater firmly adherent to the bone; arachnoid membrane transparent; vessels of the pia mater moderately full of blood; there was shrinking of the cerebral convolutions with effusion of fluid into the spaces. Substance of the brain rather soft; vessels filled with thin, pale-red, fluid blood.

The pericardium contained about three ounces of straw coloured serum; the heart was healthy.

The left lung, occupied throughout by tuberculous matter, presented in its interior a large vomica capable of holding the closed fist of a large man. This cavity, almost completely empty, and traversed by the branching remains of bronchi and blood-vessels, had opened into the sac of the pleura, and was bounded externally by five or six of the ribs, to which the remains of the lung were firmly adherent. There were several other smaller cavities, of which about three opened by rounded orifices into different parts of the pleura. The right lung in its upper three-fourths was completely consolidated by the deposit of tubercle, which had in many places softened into cavities. Only the lower fourth was fit for purposes of respiration, and tubercles, even here, were scattered rather plentifully about. The pleura pulmonalis was here and there covered by a thin layer of soft semifluid lymph. The abdominal viscera were pale and the intestinal canal was contracted: there was no fat either in the peritoneal folds or in the lumbar region. The lower part of the ilium was dark, and the walls felt thicker than natural; upon opening the tube a series of ulcers were found upon the mucous membrane, which was of deep reddish-brown hue. Some of the ulcers as large or larger than a shilling, with everted edges, had perforated the muscular coat and were bounded by the serous covering of the intestine. Others were of smaller size and not so deep; granulations had sprung up in some, giving to the surface the appearance of a healing sore. There were some ulcers along the upper part of the cœcum and colon, but the rest of the intestinal tube was healthy.

The patient, whose post-mortem examination has been here described, was an emaciated subject, who, before death, had been, apparently from mere weakness, confined to his bed. He made no complaint respecting his chest, nor was his cough sufficiently severe to distress him. He lay powerless, helpless, yet uncomplaining, until his death. Suspicions were entertained by those who attended him that there was disease of the lungs, and the large cavity, here described, was detected by auscultation; so feeble, however, was the respiration, that no very definite opinion, in the absence of all complaint from the patient, could be formed. Dr. Wood, the resident medical officer at Bethlem Hospital, informs me that he has frequently remarked the apparent exemption from suffering, in the insane, when labouring under extensive disorganization of the lungs. No symptoms indicate the amount of disease, nor does the patient express any desire to be relieved of that, which in one of sound mind would be a source of constant misery.

The effects of simple inflammation are very commonly seen in the contents of the chest. Adhesions of the pleural surfaces; the effusion of lymph, serum, or pus; congestion, softening, or consolidation of the substance of the lung; occasionally also, gangrene. The interesting point in connexion with such changes is the small amount of constitutional disturbance which they excite in proportion to their severity. Patients, the subjects of severe inflammatory diseases, have been known to lie in bed feeble, emaciated and silent, uttering no complaint which could excite the attention of those about them.

Thomas E——, aged 29, died in Bethlem Hospital, January 30, 1850. There had been no change in his symptoms before death; he sank apparently from general weakness.

There was great turgidity of the vessels of the brain and its membranes; the pia mater was gorged with blood. The cortical substance of both cerebrum and cerebellum presented a pinker tint than natural; there was congestion of the cerebral vessels, and effusion of fluid into the ventricles.

There were no adhesions of the pleuræ on either side of the chest. The posterior portion of the left lung was greatly congested with blood; though still crepitant upon pressure it was dark coloured, soft, and broke down easily under the fingers. Upon the surface of the upper lobe there was a dark black spot, without change of structure in the pleura. Upon division this was found to indicate a mass of pulmonary substance equal in size to a small orange, quite black with offensive gangrenous odour.

There were no morbid changes in any of the abdominal viscera.

A similar condition of the substance of the lung was seen in the following cases:—

*Examination of the body of W. L., March* 18, 1850.

There was general congestion of the vessels of the brain, &c. In both the lungs portions were found of the darkish colour from internal vascular congestion, and hepatized. The pulmonary substance in these hepatized portions was broken down in the centre, infiltrated with a stinking ichor, and mortified. There were seven of such portions in the left lung, the largest measuring about two inches each way: the others not larger than a walnut or filbert. The mortified parts were fewer in the right lung, of which, however, the posterior portion was more extensively hepatized.

The abdominal viscera were healthy.

*Examination of the body of A. B., June* 12, 1850.

The skull-cap was very heavy; the arachnoid membrane was transparent, but the cerebral vessels were gorged with fluid blood.

The cavities of both pleuræ were lined by a thick continuous layer of soft, yellow, recently effused fibrin; there were a few soft adhesions, and but a very small amount of sero-purulent effusion. Both lungs floated in water; but parts of their substance were softer than natural, œdematous, and infiltrated by fluid. Numerous dark spots, varying in size from a pin's head to a split bean, occupied the surface of the lungs under the pleural covering. They were surrounded by a wavy yellow line, and contained cavities filled either by pus, or by softened lung. Many of the cavities emitted the unpleasant odour of mortification, and some seemed on the point of bursting into the pleural cavity; none could be found which had absolutely given way.

The abdominal viscera were healthy.

The frequent occurrence of morbid alterations of structure in the contents of the thorax is familiar to all accustomed to open the bodies of the insane. Of 72 persons examined consecutively in Bethlem Hospital, 55 exhibited instances of pectoral disease. Dr. Webster, in a report published in the "Medical and Chirurgical Transactions," thus analyzed the cases: "43 showed either recent or old adhesions in the chest; and 31 had the lungs consolidated; in 24, suppuration had commenced; in 15 the pleura, or lungs, bore marks of previous or recent inflammation; in 12 cases there was effusion of lymph into the pleura; in 9, considerable effusion into the bronchi and air passages; in 9, the lining membrane of the trachea and bronchi was of a deep red hue." *

Death sometimes ensues from acute pericarditis—a disease of rare occurrence unconnected with rheumatism—the other viscera being sound. In a male patient, whose head, upon examination, presented the usual appearances of congestion and turgidity of the brain and its membranes, there were found traces of active and probably recent inflammation of the pericardium: both portions of the membrane were covered by a coat of fibrin of variable thickness; the loose surface was rough and shaggy; it readily peeled off from the pericardial membrane, which was found thickened, with its surface of a deepish red colour. Over a great portion of the left ventricle the heart adhered to the bag. The cavity contained about three ounces of a dull yellowish turbid fluid. Slight purulent infiltration of the cellular texture external to the pericardium at two or three points. There was an abscess on the external part of the chest towards the left side, containing a pint of thick yellow pus. It had existed for some months before death.

Such disease, if not proving fatal, terminates in adhesion, either general or partial, of the surfaces of the pericardium, or the production of those white spots so frequently met with upon the anterior surface of the right ventricle. It has been proved by my friend, Dr. Kirkes, that adhesions of the pericardium, the result of the organization of lymph, are by no means invariably permanent; that in the greater number of cases the morbid union slowly gives way, and the heart again becomes free in its fibro-serous capsule. In such cases there is no serious disturbance of the circulation. There are instances, however, of permanent adhesions of the opposed pericardial surfaces, when the ventricles, especially the left, are often found hypertrophied. In the examination of the body of a female, who died in Bethlem, with slight effusion of blood between the dura mater and arachnoid membrane, in the neighbourhood of the falx cerebri over the right hemisphere, the pericardium was found everywhere closely adherent, the adhesions being thready and areolar, and evidently of old date. There was concentric hypertrophy of the left ventricle, the walls being more than twice the natural thickness, and of deep red colour. The other viscera were healthy.

Morbid changes amongst the abdominal viscera are by no means so common as in the contents of the cranium or of the thorax. The alimentary canal and the adjacent glandular structures are usually in a healthy state. It might have been expected that the kidney would often be found diseased; and then, from the known effect of the admixture of urea with

* Transactions of Medical and Chirurgical Society. Series II. Vol. VIII.

the blood, an attempt might have been made, upon these grounds, to explain some of the morbid conditions of the brain. The contrary, however, is the fact; the capsule rarely adheres more firmly than natural to the cortical substance: there is generally the usual proportions of the two substances. Small cysts, formed by dilatations of the uriniferous tubules are occasionally met with, and in one case both kidneys were atrophied, being not above one-third the natural size.

I have seen one instance of true cystic degeneration of the kidneys; both glands being from four to five times beyond the natural size, and converted into a mass of cysts filled with fluids of different colour and consistence, varying from a light-blue to a deep brown or black hue; from the fluidity of water to the thickness and stickiness of bird-lime. But amongst these cysts, which were clearly and indisputably traced by Mr. Quekett to the uriniferous tubes, there existed much of the true glandular structure, elongated, twisted, and displaced, but in other respects healthy; so that, during life, a considerable quantity of urine flowed, and there was no suspicion of renal disease.

One cannot but imagine, in the later stages of such an affection, there must be some failure in the proper excretory power of the kidneys; some of the constituents of the urine must cease to be eliminated; yet the amount of fluid passed in these cases is rarely below, and sometimes even above, the natural standard. Rayer has related some cases of this disease as it was observed in persons of highly excitable temperament; but we cannot do more than allow that any disturbance in the function of so important an organ as the kidney, would, by its influence on the blood, indirectly affect the brain, and thus perhaps hasten the manifestation of intellectual disturbance in one previously so disposed.

In cases of tuberculosis of the lungs, the usual tuberculous ulcers are found along the course of the ilium and cæcum. In cases of typhoid fever, ulcers characteristic of that affection are met with in the same situation: the alimentary canal is sometimes perforated, when the escape of its contents leads to acute peritonitis, during which the same indifference to suffering on the part of the patient has been often observed. In the following case an ulcer has perforated the jejunum. It is related to show the extent which disease may attain in patients so circumstanced.

*Examination of John ——, Bethlem Hospital.*

There was effusion of serum into the pia mater; the vessels of the brain were full of blood; the lateral and the third ventricles were greatly enlarged; more than two ounces of fluid in the cavities. Foramen of Monro large; septum lucidum distended, thin, and had actually given way in one point, having a few separate shreds in which individual vessels were seen.

The right lung adhered strongly and universally to the cavity of the chest; there was a vast cavern in the upper lobe; the entire lower lobe tuberculated and excavated by suppurating cavities throughout. The left lung was not adherent; tuberculous masses, from the size of a pea to a gooseberry, but not yet softened, were scattered through it. Active vascular congestion in the pulmonary texture immediately surrounding these masses.

There were universal adhesions, partly ancient, partly recent, of the several abdominal viscera to each other and to the parietes. The peritoneum lining the cavity and covering the intestinal canal, and the omentum was thickened by an universal tubercular affection. The tubercles were minute, but crowded into the closest arrangement. There were partial firm adhesions of the omentum and different parts to each other and to the parietes. There had been universal and recent violent inflammation of the peritoneum, with an ulcerated aperture in the jejunum, from which the intestinal contents had escaped in small quantity. The convolutions of the intestine were closely agglutinated; there were collections of thick puriform fluid on separating them, and effusions of soft yellow lymph, from the size of a pin's-head to half-a-crown, seen in countless number. The peritoneal covering, both of the small and large intestines, was deeply discoloured in many parts by intense vascular congestion. That of the mesentery was in the same state, with the mesenteric glands slightly swollen by recent inflammation.

It must be confessed that the evidence is still unsatisfactory as to the exact nature of those conditions upon which unsoundness of mind depends; yet the reports which have been given in this journal show,—first, that the body of a lunatic is rarely, perhaps never, opened without our discovering *some morbid appearances within the cranium;* secondly, that there are frequently to be seen the traces of serious organic disease within the chest, and more rarely in the cavity of the abdomen. These morbid appearances generally indicate disease of old standing, but occasionally of more recent date.

Can we then feel surprised, even if such changes are but indirectly connected with sensorial disturbance, that, upon the return of the insane, reported cured, to society, relapses are so frequent?

